# Targeting Thymidylate Synthase Enhances the Chemosensitivity of Triple-Negative Breast Cancer Towards 5-FU-Based Combinatorial Therapy

**DOI:** 10.3389/fonc.2021.656804

**Published:** 2021-07-15

**Authors:** Nair Hariprasad Haritha, Akbar Nawab, Vinod Vijayakurup, Nikhil Ponnoor Anto, Vijayasteltar B. Liju, Vijai V. Alex, Areekkara Nisthul Amrutha, Sreekumar U. Aiswarya, Mundanattu Swetha, Balachandran S. Vinod, Sankar Sundaram, Maria V. Guijarro, Thomas Herlevich, Archana Krishna, Nesteena K. Nestory, Smitha V. Bava, Chittalakkottu Sadasivan, Maria Zajac-Kaye, Ruby John Anto

**Affiliations:** ^1^ Division of Cancer Research, Rajiv Gandhi Centre for Biotechnology, Thiruvananthapuram, India; ^2^ Department of Anatomy and Cell Biology, Cancer and Genetics Research Complex, University of Florida, Gainesville, FL, United States; ^3^ The Shraga Segal Department of Microbiology, Immunology and Genetics, Faculty of Health Sciences, Ben-Gurion University of the Negev, Beer Sheva, Israel; ^4^ Department of Biotechnology and Microbiology, Kannur University, Kannur, India; ^5^ Department of Biotechnology, University of Calicut, Malappuram, India; ^6^ Department of Pathology, Government Medical College, Kottayam, India

**Keywords:** breast cancer, thymidylate synthase, chemoresistance, chemosensitization, curcumin, 5-FU

## Abstract

**Background:**

The ongoing treatment modalities for breast cancer (BC) primarily rely on the expression status of ER, PR and HER-2 receptors in BC tissues. Our strategy of chemosensitization provides new insights to counter chemoresistance, a major obstacle that limits the benefits of chemotherapy of mammary cancers.

**Methods:**

By utilizing a murine breast cancer model employing NSG mice bearing orthotopic triple-negative breast cancer (TNBC) xenografts, we have evaluated the ability of phytochemical curcumin in chemosensitizing BC to 5-Fluorouracil (5-FU) chemotherapy and the differential modulations of cellular events in response to this strategy, independent of their receptor status.

**Results:**

A significant synergistic antitumor potential was observed in the murine model with a sub-optimal dose treatment of 5-FU plus curcumin, as evaluated by a reduction in the tumor-related parameters. We authenticated the pivotal role of thymidylate synthase (TS) in regulating the 5-FU–curcumin synergism using the TNBC pre-clinical model. Our study also confirmed the pharmacological safety of this chemotherapeutic plus phytoactive combination using acute and chronic toxicity studies in Swiss albino mice. Subsequently, the molecular docking analysis of curcumin binding to TS demonstrated the affinity of curcumin towards the cofactor-binding site of TS, rather than the substrate-binding site, where 5-FU binds. Our concomitant *in vivo* and *in silico* evidence substantiates the superior therapeutic index of this combination.

**Conclusion:**

This is the first-ever pre-clinical study portraying TS as the critical target of combinatorial therapy for mammary carcinomas and therefore we recommend its clinical validation, especially in TNBC patients, who currently have limited therapeutic options.

## Introduction

The highly heterogeneous nature of breast cancer (BC) is a major hurdle in developing single mode of treatment against diverse BC subtypes. Luminal tumors respond moderately well to hormonal interventions while human epidermal growth factor receptor-2 positive (HER-2+) tumors can effectively be managed using a diverse array of anti-HER-2 therapies. Despite this improvement in BC treatment modality, only 20% of triple-negative breast cancer (TNBC) and basal-like breast cancer (BLBC), devoid of estrogen (ER), progesterone (PR) and HER-2 receptors, respond well to standard chemotherapy, while the occurrence of these subtypes of BC is increasing in an alarming rate, worldwide (1–3).

5-Fluorouracil (5-FU) is the first rationally designed anti-metabolite administered against a variety of solid tumors including BC, where the drug inhibits thymidylate synthase (TS), the key enzyme for *de novo* synthesis of 2’-deoxythymidine-5’-monophosphate (dTMP) (4). Though 5-FU is not an exception to the severe side effects induced during the treatment (5), its peculiarity to target TS expression independent of BC receptor status and the cost-effectiveness compared to other chemotherapeutics (6, 7), make it an absolute choice for chemotherapy for BC. However, the use of 5-FU in clinical settings is limited due to the emergence of acquired or inherent chemoresistance, an event that is mechanistically explained as a drug-induced up-regulation of its therapeutic target, TS (4). TS status is pointed out as the determining factor for the success of 5-FU chemotherapy (8–10), while other reports suggest a dual role for TS to act as an oncogene (11–13). Apart from TS, drug-induced activation of major survival signals like NF-κB, Akt and MAPKs have also been demonstrated as major players responsible for 5-FU chemoresistance (14–17).

Curcumin, a bioactive from *Curcuma longa* is an extensively studied phytochemical owing to its various therapeutic utilities *in vitro* and *in vivo*. Numerous reports, including that of ours, have profoundly established the anti-proliferative, anti-angiogenic, anti-metastatic and pro-apoptotic properties of curcumin (18–24). We have also reported several studies demonstrating the synergistic cytotoxic effect of curcumin with conventional chemotherapeutics (20, 24, 25), including an *in vitro* study (15), which identified a synergistic cytotoxic combination of 5-FU and curcumin, effective in different BC cell lines, irrespective of their receptor status. In this study, we tested multiple dosage combinations of 5-FU (1, 2.5, 5 &10 µM) and curcumin (2.5, 5 & 10 µM) in BC lines of varying receptor status and noted that a combination of 10 µM 5-FU and 10 µM curcumin could induce synergistic cytotoxicity *in vitro*, in all the cells studied, observing maximum cytotoxicity in the TNBC cell line, MDA-MB-231. This report, for the first time, provided a mechanistic explanation for 5-FU-curcumin synergism in BC cells (15). Though this combinatorial effect has also been reported in gastric cancer cells (26) and colon carcinoma (27–30), no study to date, authenticates the therapeutic efficacy of the combination *in vivo*, with mechanism-based evidence.

The ability of this combination to overcome the undesirable effects of 5-FU chemotherapy by curcumin-mediated chemosensitization of BC *in vitro* through down-regulation of the key cell survival pathways, prompted us to translate it to an *in vivo* system for proper pre-clinical validation. Since attaining a serum concentration of 10 µM curcumin is practically impossible *in vivo*, we conducted a pilot study in Nonobese Diabetic/Severe Combined Immunodeficiency gamma (NSG) mice with 2 drug combinations i.e., 10 µM 5-FU with either 10 µM or 5 µM curcumin. Notably, the combination with 5 µM curcumin was equally effective as the other, though the former combination did not show a synergism *in vitro*. This prompted us to continue with this particular dosage which also stays approximate to the maximal bioavailable concentration of the curcumin. Hence, we utilized a human TNBC xenograft model in NSG mice, which explicitly demonstrated the superior anti-tumor effect of the 5-FU-curcumin combination, which specifically leads to the down-regulation of TS pathway. Furthermore, our precise studies in the TNBC model corroborated the role of TS to serve as the key regulator of this synergism. Our subsequent molecular docking studies showed the interaction site of curcumin on TS and explained its affinity towards TS alone or in the presence of FdUMP, the active metabolite of 5-FU. Hence, this is the first pre-clinical study to date that portrays TS as a promising clinical target for curcumin-mediated chemosensitization of mammary tumors towards 5-FU chemotherapy, with conclusive *in vivo* and *in silico* evidence.

## Methods

### Cell Lines

The breast cancer cell line MDA-MB-231 and human embryonic kidney cells 293T (HEK293T) were purchased from ATCC and maintained in appropriate medium. Mycoplasma tests were performed on parent cell lines and stable cell lines every 6 months using PCR method.

### Reagents and Antibodies

5-FU was purchased from Calbiochem (San Diego, CA, USA). Curcumin, X-treme GENE™ HP DNA transfection reagent and antibody against vinculin were procured from Sigma-Aldrich (St. Louis, MO, USA). Antibodies against p-ERK1/2, p-p38, p-JNK, p-Akt, p-IKK, c-Myc and caspases were obtained from Cell Signaling Technology (Beverly, MA, USA). Antibodies against p-p65, MDR-1, VEGF, Bcl-2, cIAP1, XIAP, survivin, TS, PARP, Cox-2 and GAPDH were purchased from Santa Cruz Biotechnology (Santa Cruz, CA, USA). Antibody against ABCG2 was purchased from Abcam (Cambridge, Mass, USA).

### Animal Experiments

#### Orthotopic Xenograft Model of Human Breast Cancer

Xenograft studies were conducted according to protocol #RD3585, under the approval of the University of Florida Institutional Animal Care and Use Committee in accordance with federal, state and local guidelines. 6-8 weeks old, female, nulliparous mice of the strain NOD/SCID (Nonobese Diabetic/Severe Combined Immunodeficiency) gamma, the NOD.Cg-PrkdcscidIl2rgtm1Wjl/SzJ mice, commonly known as NSG mice, underwent orthotopic, mammary fat pad injection of 4x10^6^ MDA-MB-231 cells in PBS. Tumor growth was monitored by palpitation twice a week. Palpable tumors were identified 15 days post-injection and the animals were randomly assigned to four groups (n=5/group). Treatments were started from the 16th day of injection. Group1 was treated with vehicle alone, Group II received an intraperitoneal injection of liposomal curcumin at 25 mg/kg body weight on alternate days, Group III received an intraperitoneal injection of 5-FU dissolved in PBS at a dose of 20 mg/kg body weight twice weekly and Group IV received both 5-FU and curcumin. Curcumin was encapsulated in unilamellar liposome formulation made of phosphatidylcholine and cholesterol. Animals in group IV received a single intraperitoneal injection of liposomal curcumin at 25 mg/kg body weight as a pre-treatment and after 6 h, these animals again received IP injections of liposomal curcumin at 25 mg/kg (as the maximum retention time of curcumin within the body is 3 h), along with 5-FU dissolved in PBS at a dose of 20 mg/kg body weight. Drug treatment was continued up to 6 weeks, animals were euthanized and the tissue samples were collected for further analyses.

### Drug Regimen

Group I- IP injection of the vehicle (liposome) alone (on alternate days).

Group II- IP injection of liposomal curcumin 25 mg/kg body weight (on alternate days).

Group III- IP injection of 5-FU 20 mg/kg body weight dissolved in PBS (twice weekly).

Group IV- IP injection of liposomal curcumin 25 mg/kg body weight (on alternate days).

On the days of treatment with the combination of curcumin and 5-FU, the animals received,

a) IP injection of liposomal curcumin 25 mg/kg body weight (pre-treatment).
b) 6 h post first curcumin injection, two separate IP injections of liposomal curcumin 25 mg/kg body weight and 5-FU 20 mg/kg body weight dissolved in PBS, respectively (twice weekly).

### Lentiviral TS shRNA Transduction in MDA-MB-231 Cells

To evaluate the regulatory role of TS in the synergism, MDA-MB-231 cells were infected with lentiviral TS shRNA and injected orthotopically to the mammary fat pad of NSG mice.

#### shRNA Design and Lentivirus Plasmids Used

Lentiviral shRNA vector pLKO.1 (designated #89- obtained from Addgene), lentiviral TS shRNA vector (designated #64-obtained from Sigma, TRCN0000045667), target sequence: 5′CCGGCTTTGGGAGATGCACATATTTCTCGAGAAATA TGTGCATCTCCCAAAGTTTTTG-3′and lentiviral pSMPUW-GFP vector (designated #76-from Cell Biolabs, Inc) were used to produce recombinant lentivirus in HEK293T cells.

Below mentioned are the other lentiviral TS shRNA vectors obtained from Sigma and screened for the study:

5′CCGGCAGGTGACTTTATACACACTTCTCGAGAAGTG TGTATAAAGTCACCTGTTTTTG-3′ (TRCN0000045664),
5′CCGGGCAAAGAGTGATTGACACCATCTCGAGATGGT GTCAATCACTCTTTGCTTTTTG-3′ (TRCN0000045666)
5′CCGGGCTGACAACCAAACGTGTGTTCTCGAGAACACA CGTTTGGTTGTCAGCTTTTTG-3′ (TRCN0000045665)
5′CCGGCCCTGACGACAGAAGAATCATCTCGAGATGATT CTTCTGTCGTCAGGGTTTTTG-3′ (TRCN0000045663)

#### Lentivirus Production

5x10^6^ HEK 293T cells plated in to 150 mm dishes were transfected with 10µg of TS shRNA plasmid, pLKO.1 vector or pSMPUW-GFP, 5µg psPAX (lentiviral packaging plasmid) and 5µg pMD2.G (VSV-G envelope expressing plasmid) with Fugene transfection Reagent (Qiagen), according to manufacturer’s instructions. 48 h post-transfection, media containing the lentiviral particles were harvested and centrifuged at 3000 rpm at 4°C for 10 min. The supernatant was collected, mixed with 1/3rd volume of fresh media and 1:1000µl polybrene was added.

#### Transduction

5x10^6^ MDA-MB-231 cells were plated to 150 mm dishes, incubated overnight and transduced with the lentivirus for 8 h. After incubation, the infection mixture was replaced with fresh media and the lentiviral particles containing media was discarded. This procedure was repeated twice. The efficiency of transfection in HEK 293T and transduction in MDA-MB-231 was assessed using a microscope and photomicrographs were taken. Transduction efficiency was further validated through immunoblotting analysis.

### Orthotopic Xenograft Model of Human Breast Cancer Using Established MDA-MB-231^TS^ and MDA-MB-231^TS-^ Cells

6-8 weeks old, female, nulliparous NSG mice (n=40) were used for the experiment. The animals were separated into two groups. Group A and B underwent orthotopic, mammary fat pad injection of 4x10^6^ MDA-MB-231^TS^/MDA-MB-231^TS-^cells in PBS, respectively. In both groups, tumor growth was monitored by palpitation twice a week. Palpable tumors were identified 15 days-post-injection and the animals in both groups were randomly assigned to four sub-groups (n=5/sub-group). Treatments were started from the 16^th^ day of injection. Sub-group I of group A and B was treated with the vehicle alone, sub-group II of group A and B received an intraperitoneal injection of liposomal curcumin at 25 mg/kg body weight on alternate days, sub-group III of group A and B received an intraperitoneal injection of 5-FU dissolved in PBS at a dose of 20 mg/kg body weight twice weekly and sub-group IV of group A and B received both 5-FU and curcumin. Animals in sub-group IV of groups A and B received a single intraperitoneal injection of liposomal curcumin at 25 mg/kg body weight as a pre-treatment 6 h before administration of combination and these animals again received IP injections of liposomal curcumin at 25 mg/kg along with 5-FU dissolved in PBS at a dose of 20 mg/kg body weight, since the maximum retention time of curcumin within the body is 3 h. Drug treatment was continued up to 6 weeks, animals were euthanized and the tissue samples were collected for further analyses.

### Toxicological Analyses

The toxicological analysis of the combination was performed in 6-8 weeks old female Swiss albino mice as per protocol (IAEC/230/RUBY) approved by the Institutional Animal Ethics Committee, Rajiv Gandhi Centre for Biotechnology.

Acute toxicity: Doses of 0, 25 and 50 mg/kg of curcumin and 20 mg/kg of 5-FU were given to groups of six mice each. Animals were euthanized on day 8. The liver tissue was analyzed by histopathology using H&E staining and the serum was used to perform Liver Function Test (31).

Sub-chronic Toxicity: Doses of 25 and 50 mg/kg of curcumin and 20 mg/kg of 5-FU were given to groups of six mice each. Animals were euthanized after 90 days and toxicity was measured as described (31).

### Histology and Immunohistochemistry

The tumor and liver tissues from mice and rats were fixed and cryosectioned. Immunostaining of specific proteins in the tissue sections was done using Poly Excel HRP/DAB detection system universal kit for mouse and rabbit primary antibodies (PathnSitu Biotechnologies Pvt. Ltd, India) as per manufacturer’s protocol. All the immunohistochemistry images were taken in DMi8 Inverted Fluorescence Research Microscope with DMC 2900 Digital Camera.

### TUNEL Assay

TUNEL assay was performed to detect apoptosis in formalin fixed, paraffin-embedded xenograft tumor tissue sections using DeadEnd Colorimetric TUNEL System (Promega) following the manufacturer’s instructions (24).

### Western Blot Analysis

Cytoplasmic and nuclear proteins were isolated from tissue samples and were subjected to Western blotting as described earlier (24). The quantification of immunoblots was carried out using ImageJ software.

### Molecular Docking Studies

The computational studies were carried out using the software package of Schrödinger (Schrödinger LLC, New York, NY, 2018). The crystal structure of TS was retrieved from Protein Data Bank and used as the initial structure for modeling studies (PDB ID: 1JU6). The atomic coordinates of the ligands, curcumin and 5-fluoro-dUMP (FdUMP) were downloaded from PubChem (CID: 969516) and PDB (PDB ID: 1TLS) respectively. The structure correction of 1JU6 was performed with the module Protein Preparation Wizard. The crystallographic water molecules were removed and polar hydrogens were added. Energy minimization was performed up to an RMSD of 0.3 Å. A 36 Å x36 Å x36 Å receptor grid was generated, that encompasses the binding sites for the substrate and cofactor. The ligands were prepared before docking using the module Ligprep. The ligands were docked onto protein binding sites using Extra Precision (XP) mode of flexible docking (Glide, Schrödinger, LLC, New York, NY, 2018).

### Statistical Analysis

All data were expressed as mean ± SD. Data were analyzed using Prism 6.0 (GraphPad Software). One-way ANOVA measured statistical significance between the conditions. ***P-values ≤0.001, **P-values ≤0.01 and *P-values ≤0.05; ns represents non-significance.

## Results

### Curcumin Enhances the Anticancer Efficacy of 5-FU Against Orthotopically Implanted Human BC Xenografts in NSG Mice

**Figure 1:**
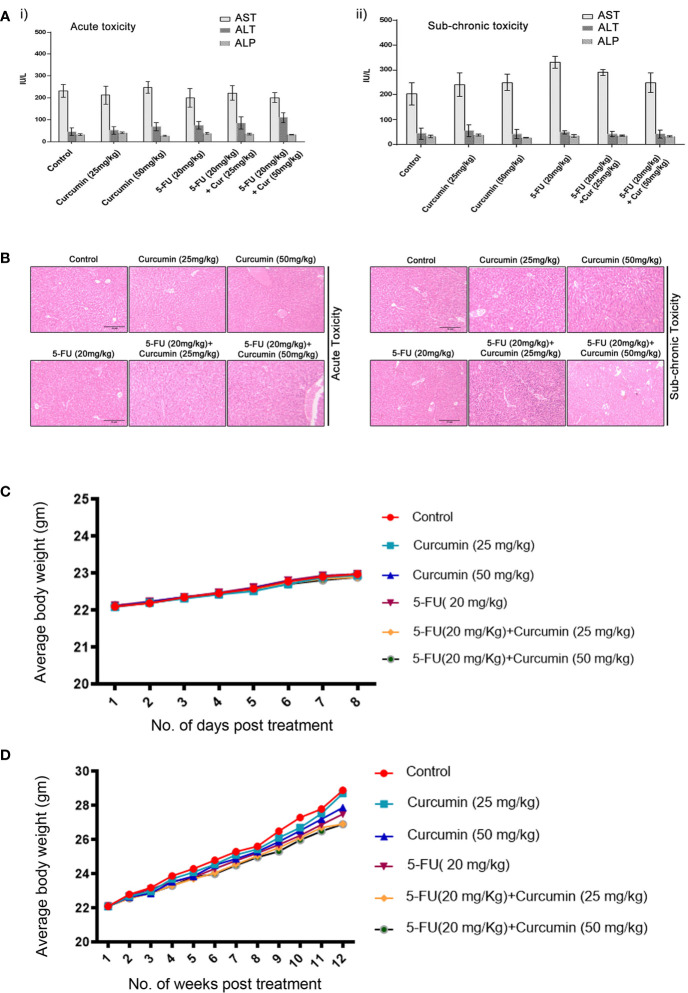
Toxicological analyses of curcumin and 5-FU alone or in combination using Swiss albino mice. [**A** (i, ii)]. Curcumin and 5-FU alone and/or in combination at the given dose, does not induce any liver cytotoxicity in both acute and sub-chronic toxicity models as assessed by biochemical analysis. i) Serum levels of AST, ALT and ALP in acute toxicity study. ii) Serum levels of AST, ALT and ALP in sub-chronic toxicity study. **(B)** Histopathological analysis of liver tissues from acute and sub-chronic toxicity study. **(C)** The average body weight of animals in different groups during the acute toxicity study. **(D)** The average body weight of animals in different groups during the sub-chronic toxicity study.

**Figure 2:**
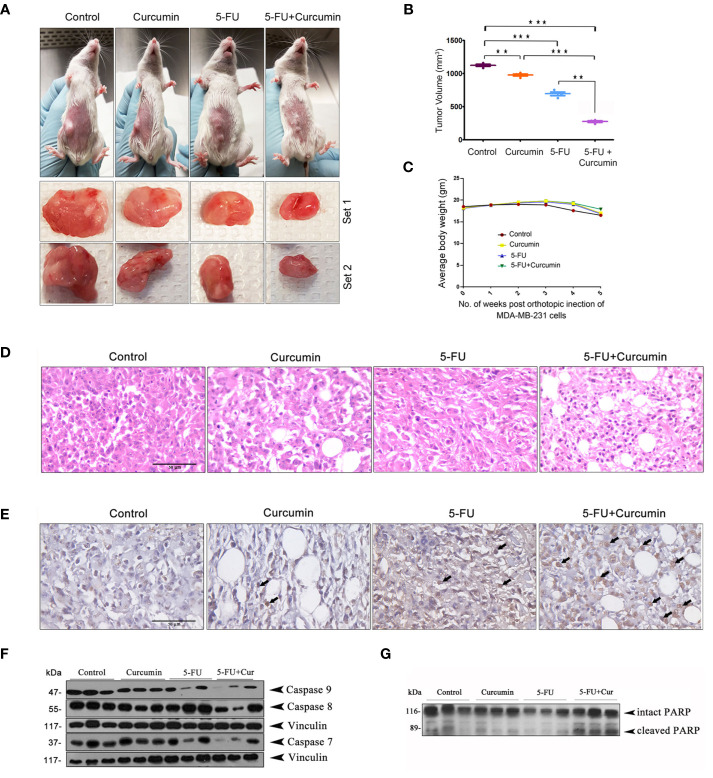
Curcumin enhances antitumor effect of 5-FU in orthotopic xenografts of human BC in NSG mice **(A)** Representative images of animals from each experimental group showing differences in tumor volume upon completion of treatment. **(B)** The mean tumor volume (in mm3) among different experimental groups is shown. Data represent two independent sets of experiments and results are shown as the mean ± S.D. P-values were calculated with one-way ANOVA. ***P-values ≤0.001 and **P-values ≤0.01. **(C)** The effect of the treatment on the average body weight of animals in different groups during the study. **(D)** Histological changes induced by the treatments, either alone or in combination are shown. **(E)** Representative photomicrographs showing TUNEL staining pattern in xenograft tumor sections. Dark brown staining is indicative of nuclei with fragmented DNA. **(F)** Curcumin potentiates 5-FU-induced cleavage of caspases in human breast cancer xenograft treated with 5-FU + Cur. **(G)** Curcumin pre-treatment markedly increased 5-FU-induced PARP cleavage in the animals treated with the combination. Total proteins were extracted from tumor samples of different experimental groups as described in Methods and subjected to Western blot analysis using specific antibodies against caspases 9, 8, 7 and PARP. All experiments were repeated thrice with samples from different animals of the same treatment groups. Vinculin levels are shown to monitor equal loading of samples.

To validate our previous *in vitro* findings (15), firstly we utilized an orthotopic xenograft model of human BC in NSG mice, established using the TNBC cell line, MDA-MB-231. The drug treatment regimen including that of the combination is as described in Materials and Methods. We previously reported the maximum retention time of liposomal curcumin as 3 h (25), hence a second dose of curcumin was given along with 5-FU to maintain the presence of curcumin in the mouse system. In order to evaluate the pharmacological safety of the combination, a detailed toxicological analysis was conducted in Swiss albino mice using acute and sub-chronic toxicity studies (**Figures 1A, B**). The animals did not exhibit any abnormal behaviour and did not show any significant deviation from the normal reference range of serum AST (Aspartate aminotransferase), ALT (Alanine transferase) and ALP (Alkaline phosphatase). Even though, in the acute toxicity study, the group of mice, which received 20 mg/kg 5-FU+ 25 mg/kg of curcumin and the group which received 20 mg/kg 5-FU+ 50 mg/kg of curcumin showed a slight increase in ALT level in comparison to other groups (**Figure 1A**), the changes fell in the normal reference range indicating safety of the regimen. The histopathological evaluation of the liver tissue did not show any toxic changes or cholestasis/necro-inflammatory reactions (**Figure 1B**) attesting the pharmacological safety of the combination. Moreover, the treatments did not induce any significant effect on the body weight of the animals also (**Figures 1C, D**). The mean tumor volume of the group treated with 5-FU+Cur was remarkably diminished compared to that of the individual treatment groups (**Figure 2A**). The animals treated with the combination (5-FU+Cur) exhibited a significant reduction (~5 fold) in their tumor sizes in comparison to the control, at the time of necropsy (**Figure 2B**). However, animals treated with curcumin alone had a minimal reduction (~1.25 fold) in tumor size and volume whereas treatment with 5-FU alone produced a significant reduction (~2.5 fold) confirming the synergistic antitumor efficacy of the combination (**Figure 2B**). **Figure 2C** shows the changes in the average body weight of the animals in different groups during the course of the treatment. Presence of increased number of apoptotic cells, apoptotic bodies and micronuclei in the histopathological evaluation of tumor sections showed that curcumin accelerates 5-FU-mediated apoptosis (**Figure 2D**). Curcumin-mediated enhancement of 5-FU-induced apoptosis in the tumor tissues was confirmed using TUNEL staining (**Figure 2D**), where the sections from animals treated with curcumin alone did not show significantly positive apoptotic cells compared to tissues treated with 5-FU alone, while in the combination, curcumin augmented the apoptotic effects of 5-FU (**Figure 2E**). A significant increase in the percentage of apoptotic cells in the sections obtained from animals treated with 5-FU+Cur attest curcumin-mediated enhancement of 5-FU-induced apoptosis (**Supplementary Figures 1A, B**). The combination also displayed a notable enhancement in the cleavage of the caspases 9, 8 and 7 (**Figure 2F**). An elevation in apoptosis induced by 5-FU+Cur was confirmed by an increase in PARP cleavage (**Figure 2G**).

### The Antitumor Potential of 5-FU Against TNBC Was Augmented by Curcumin *via* Repression of TS and Key Anti-Apoptotic Factors

**Figure 3:**
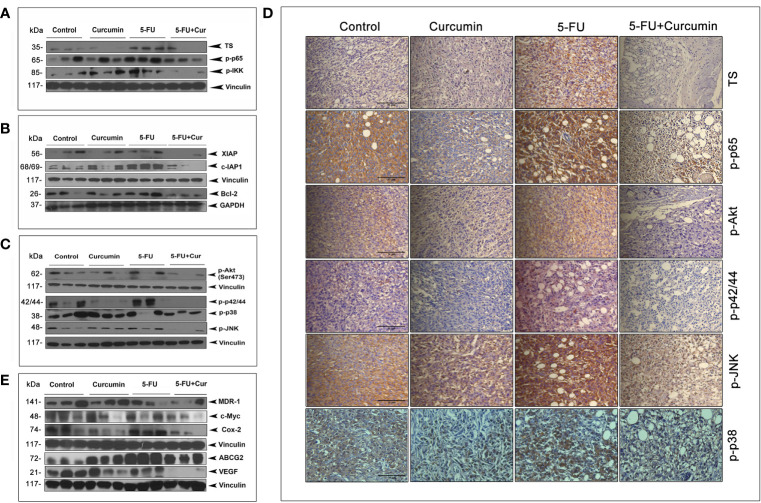
Curcumin attenuates 5-FU-induced over-expression of TS together with activation of NF-κB and other major survival signals. **(A)** Treatment with 5-FU alone induced a significant over-expression of TS and activation of NF-κB and IKK, which was remarkably down-regulated by curcumin pre-treatment. Total proteins were extracted from tumor samples and were subjected to Western blot analysis using specific antibodies against TS, p-p65, and p-IKK. **(B)** Curcumin pre-treatment down-regulates 5-FU induced up-regulation of XIAP, c-IAP1, and Bcl-2. All experiments were repeated thrice with samples from different animals of the same treatment groups. Vinculin and GAPDH levels demonstrate equal loading of samples. **(C)** Curcumin pre-treatment remarkably suppressed 5-FU-induced activation of Akt and MAPKs. **(D)** Immunohistochemical analysis of the expression status of TS and p65 subunit of NF-κB, p-Akt, p-p42/44 and p-JNK in tumor tissue sections from experimental groups. **(E)** Curcumin pre-treatment down-regulates 5-FU-induced over-expression of MDR-1, c-Myc, Cox-2, ABCG2 and VEGF in human BC xenografts. All experiments were repeated thrice with samples from different animals of the same treatment groups. Anti-Vinculin immunoblot serves as the loading control.

We had previously pinpointed TS-dependent down-regulation of NF-κB as the pivotal event regulating the 5-FU-Cur synergistic effect, *in vitro* (15). To outline a mechanistic explanation for the 5-FU-Cur synergism *in vivo*, we studied the expression status of TS and p65 subunit of NF-κB in all the experimental groups of TNBC xenografts. We observed a hike in the expression of TS (2 out of 3) and phospho p65 (3 out of 3) in the 5-FU alone group compared to control and curcumin alone. Supporting our *in vitro* data, curcumin pre-treatment produced a drastic reduction in 5-FU-induced over-expression of TS and activation of NF-κB *in vivo*, which was authenticated by the minimal expression of IKK phosphorylation in the group treated with the combination (**Figure 3A**). 5-FU-induced up-regulation of various members of the IAP family contribute to chemoresistance (15) and are trans-activated in tumor cells as a part of the NF-κB-mediated cell survival pathway (18). Our immunoblot analysis indicated an over-expression of XIAP, c-IAP1, and Bcl-2 (from the IAP family) in tissue lysates isolated from animals treated with 5-FU alone while the expression levels of these molecules were attenuated in the animals treated with 5-FU+Cur (**Figure 3B**).

Immunoblot studies also show that curcumin pre-treatment can successfully reduce 5-FU-induced activation of Akt and all the MAPKs (**Figure 3C**). IHC analysis of the expression status of TS, p-p65, p-Akt, p-p42/44, p-JNK and p-p38 in tumor tissue sections from different experimental groups also confirms the same (**Figure 3D**). Several downstream effectors of the NF-κB pathway has been reported to have a role in BC incidence and progression, such as MDR-1 (32), c-Myc (33), Cox-2 (34), BCRP/ABCG2 (35) and VEGF, which are involved in regulating the response to chemotherapy, cellular growth, proliferation, and transmigration, respectively. Our observation of the ability of curcumin to down-regulate 5-FU-induced activation of NF-κB urged us to analyze the expression status of these molecules in our treatment regimens. The general immunoblot pattern indicates that curcumin can successfully down-regulate the basal as well as 5-FU-induced expression of the above mentioned NF-κB downstream effector molecules in the animals treated with 5-FU+Cur (**Figure 3E**).

### TS Serve as the Critical Regulator in the Synergistic Antitumor Effect of 5-FU and Curcumin

**Figure 4:**
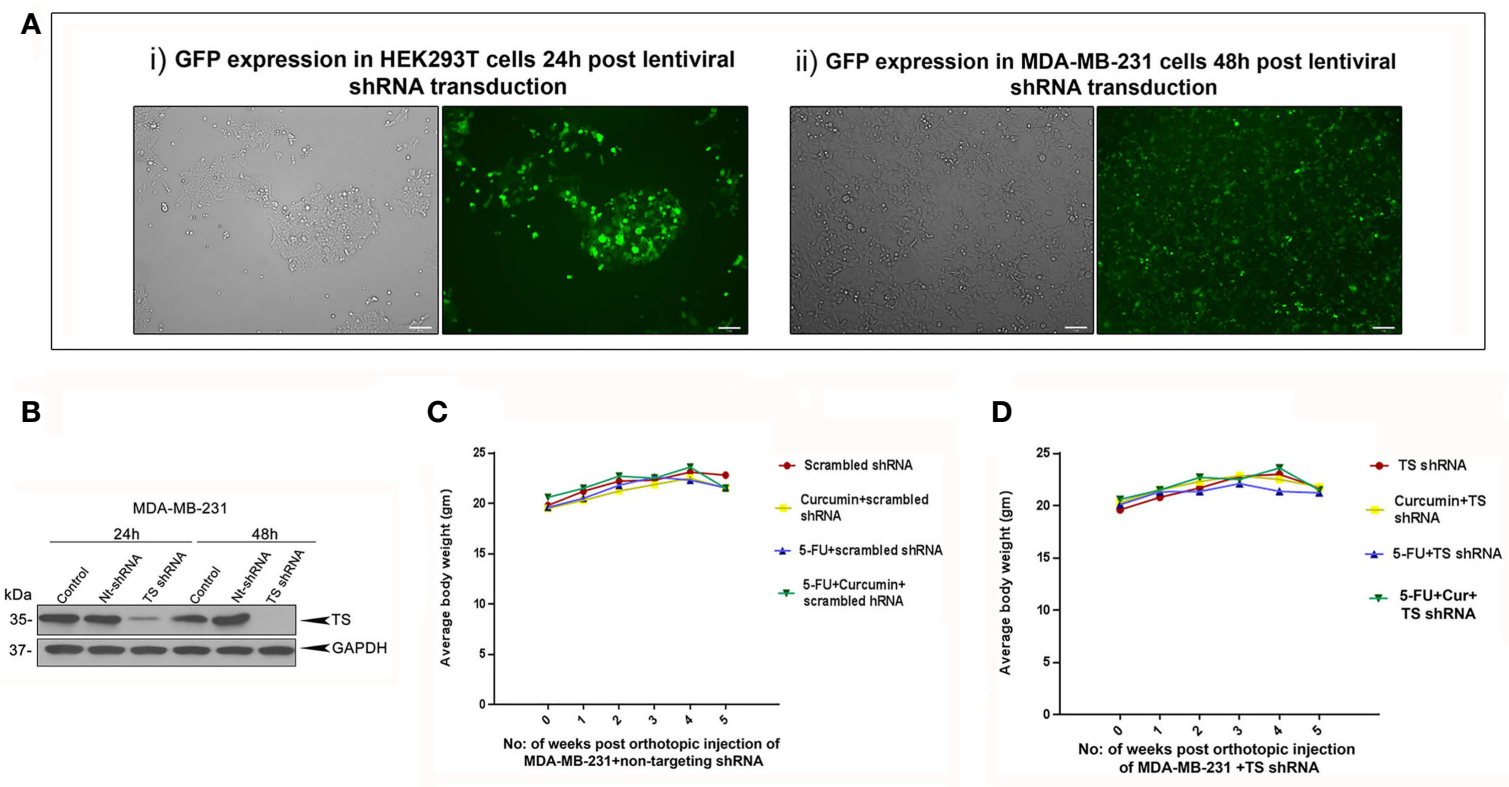
Analysis of the efficiency of lentiviral transduction and expression status of TS in transduced cells. **(A)** Efficiency of lentiviral transfection in HEK 293T cells and successive infection in MDA-MB-231 cells, respectively as assessed by GFP expression. **(B)** Time-dependent analysis of expression status of TS in MDA-MB-231 cells transduced with TS shRNA compared to that of untreated control and cells transduced with control/non-targeting shRNA (Nt-shRNA) using immunoblotting. GAPDH levels are shown as the loading control. **(C, D)** Comparison of body weight from different treatment groups bearing MDA-MB-231^TS^ xenografts and MDA-MB-231^TS-^ xenografts, respectively.

**Figure 5:**
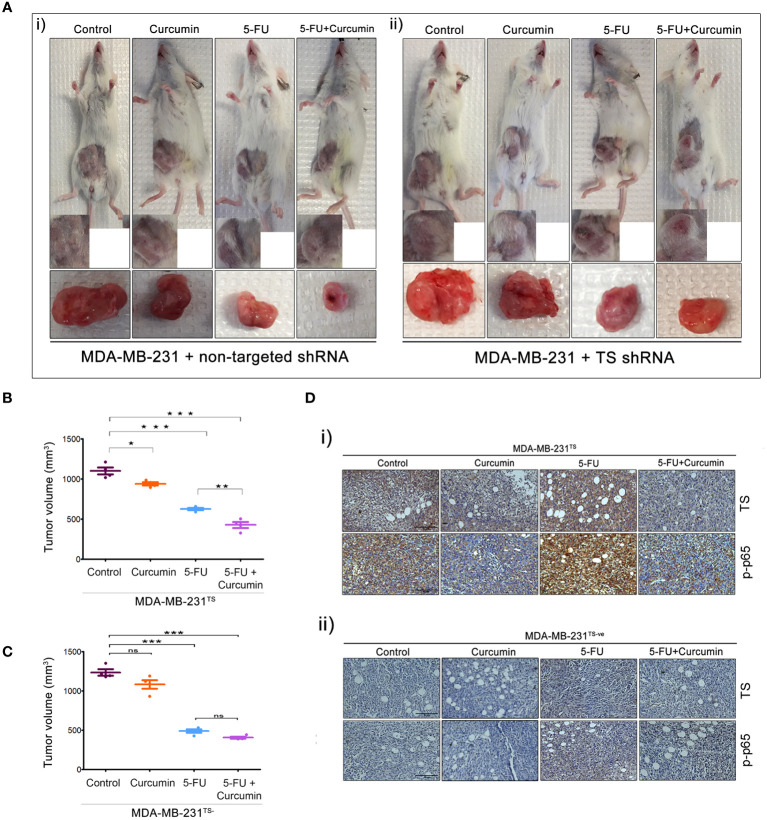
The role of TS in regulating the synergism of 5-FU and curcumin. [**A** (i, ii)] Representative images of animals from each experimental group, bearing tumor produced by orthotopic injection of both sets of transduced cells and tumors excised showed a reduction in tumor size upon completion of treatment. **(B, C)** Graphs showing a comparison of tumor volume of MDA-MB-231^TS^ and MDA-MB-231^TS-^ xenografts, respectively upon completion of treatment. Significant reduction in tumor volume is observed in animals bearing MDA-MB-231^TS^ xenografts, upon treatment with combination while no significant reduction in tumor volume is observed in animals bearing MDA-MB-231^TS-^ xenografts. Data represent two independent sets of experiments and results are shown as the mean ± S.D. P-values were calculated with one-way ANOVA. ***P-values ≤0.001, **P-values ≤0.01 and *P-values ≤0.05; ns represents non-significance. [**D** (i, ii)] Immunohistochemical analysis of expression status of TS and p65 sub-unit of NF-κB in different treatment groups of MDA-MB-231^TS^ and MDA-MB-231^TS-^ xenografts, respectively.

The striking analogy between our previous *in vitro* (15) and the current *in vivo* observations emphasizes a determining role for TS in regulating the synergism of 5-FU and curcumin. To authenticate this, we generated MDA-MB-231^TS^ and MDA-MB-231^TS-^ cells that stably express control/non-targeting shRNA and TS shRNA respectively, by transducing parental MDA-MB-231 cells (**Figure 4A**). The expression levels of TS in these cells were monitored using immunoblotting (**Figure 4B**) and the corresponding orthotopic BC xenografts were developed in NSG mice thereafter. Comparative analysis of the body weights of mice did not show any significant variation (**Figures 4C, D**). While the combination of 5-FU+Cur exhibited a significant attenuation in tumor volume in animals bearing MDA-MB-231^TS^ xenografts (**Figures 5A**-i**, B**), which were in complete concordance with the previous results (**Figure 2B**), the combination was unsuccessful in eliciting any significant effect on tumor volume in mice harboring MDA-MB-231^TS-^ xenografts, compared to mice treated with 5-FU alone, emphasizing the role of TS as a critical factor in mediating the synergistic effect (**Figures 5A**-ii**, C**). **Supplementary Figures 1C, D** shows the average tumor volume of animals in different treatment groups from the starting till the end of the study. The results also demonstrate that curcumin fails to chemosensitize TS-deficient MDA-MB-231 cells to 5-FU therapy (**Figures 5A**-ii****). IHC studies of TS and NF-κB in the tumor samples derived from animals bearing MDA-MB-231^TS^/MDA-MB-231^TS-^ xenografts confirmed TS-dependent down-regulation of NF-κB (**Figures 5Di**-ii****).

**Figure 6:**
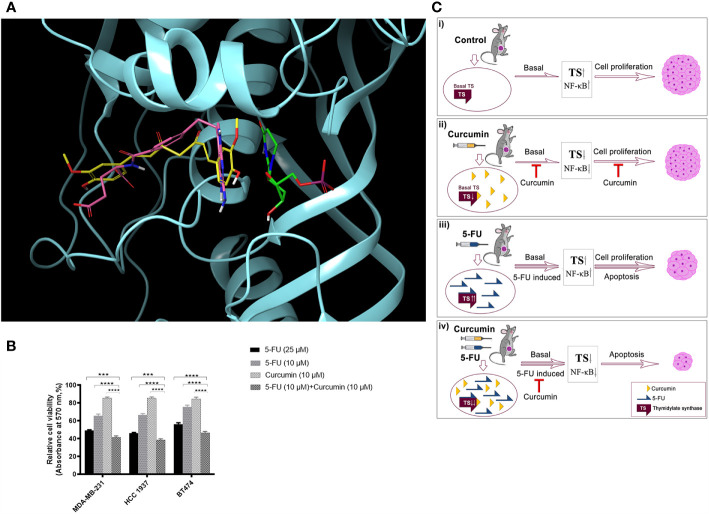
Docking analysis of curcumin binding on TS **(A)** Docking analysis of curcumin binding on TS. Comparative binding pose of Curcumin (yellow), LY231514 (pink), and FdUMP (green) at the catalytic center of TS generated by molecular docking studies. Curcumin and LY231514 span the cofactor binding site of TS whereas FdUMP binds the substrate binding site. Protein is displayed in cartoon style and ligands are represented as tubes. **(B)** Effect of 5-FU and curcumin, alone or in combination, on breast cancer cells of different receptor status. A total of 5000 cells in triplicates were exposed to the indicated concentrations of the drugs for 48 h and subjected to 3-(4,5-dimethylthiazol-2-yl)-2,5-diphenyltetrazolium bromide (MTT) assay. Relative cell viability was determined as percentage absorbance over untreated control. Data represent three independent sets of experiments and results are shown as the mean ± S.D. **** and *** represents P-values ≤0.0001 and ≤0.001 respectively. **(C)** A schematic representation of 5-FU-curcumin synergism against breast tumor progression. i The mammary tumors induced in NSG mice exhibit an up-regulation of key anti-apoptotic factors like TS and NF-κB, which promotes cell proliferation leading to BC progression. ii Phytochemical curcumin impedes the activation of these factors by binding to TS and thereby chemosensitizing the tumor cells making it susceptible to programmed cell death. iii 5-FU, the widely used anti-cancer drug, elicits significant apoptosis of tumors cells by its mechanism of action though in parallel it elevates the activation of various survival signals and key cell proliferative factors including TS enzyme causing drug-induced chemoresistance as a side-effect. iv The combinatorial administration of curcumin along with 5-FU attenuates the drug-induced augmentation of TS and the downstream anti-apoptotic regulators that promote chemoresistance and subsequently lead to cell death resulting in drastic tumor regression and thereby functions as an efficient therapeutic regimen against all BC types irrespective of their receptor status.

Molecular-docking was performed to analyse the interactions between 5-FU-Cur and the effector molecule (TS). The catalytic centre of TS possesses substrate and cofactor (folate) binding sites. A receptor grid, which encompasses both sites, was generated and curcumin was docked on to this grid. Curcumin occupied the TS cofactor binding site with Glide score -7.07 kcal/mol whereas the crystallographic antifolate inhibitor LY231514 (Pemetrexed, Alimta^®^) scored -7.70 kcal/mol. Both curcumin and LY231514 made hydrogen bonds with residue A312. Interestingly, curcumin made additional hydrogen bonds with residues R50 and R78. The interactions with A312 and R50 anchor curcumin deep within the folate binding site. The similar binding affinity of curcumin and LY231514 towards the TS cofactor binding site indicates the efficacy of curcumin to function as a cofactor site-binding inhibitor of TS. 5-fluoro-dUMP (FdUMP), the active metabolite of 5FU, forms a covalent adduct with TS *in vivo* (36). This prompted us to dock this molecule on to TS active site where it occupied the substrate-binding site with Glide score -5.74 kcal/mol. **Figure 6A** illustrates the comparative binding pose of curcumin, LY231514 and FdUMP on TS. To confirm that the findings our study is not confined to the TNBC cell line, MDA-MB-231 and will work independent of the receptor status, we evaluated the synergistic effect of the combination in another TNBC cell line, HCC 1937 and triple positive cell line, BT474. The results obtained were in concordance with that obtained in MDA-MB-231 (**Figure 6B**). Hence, through this study, we propose a novel and effective chemotherapeutic regimen against BCs of all receptor status (**Figure 6C**).

## Discussion

The evasion of programmed cell death is a major hallmark that flags drug-induced chemoresistance in neoplastic cells. Breast cancer, the most diversified amongst all cancers, lacks a unique way of therapy due to varying molecular subtypes. The present study, using orthotopic xenograft model of human TNBC, has demonstrated the ability of the phytoactive curcumin in chemosensitizing BCs, irrespective of their receptor status, by contributing synergistically to the chemotherapeutic efficacy of 5-FU. Also, the studies performed in the substantiated TS as the principal target for this combinatorial chemotherapeutic regimen.

Although the phytoproduct curcumin has been established as an ideal chemopreventive and chemosensitizer, which influence multiple signaling pathways that promote chemoresistance (18, 21), its clinical trials as a chemotherapeutic have not been favorable owing to the poor bioavailability of this compound (37). Bolstered by our previous *in vitro* studies, where we established curcumin as an effective chemosensitizer towards 5-FU treatment (20, 24), we conducted the current study to verify these results *in vivo*, using a bioavailable dose of curcumin and to unravel the underlying mechanisms of this synergism. An explicit synergistic antineoplastic effect was observed with a combination of doses equivalent to 10µM 5-FU and 5µM curcumin, as assessed by reduction in the tumor volume and size which indicates an increase in apoptotic tumor cells. The utilization of MDA-MB-231, a TNBC cell line, in our xenograft experiments expelled the possibility of any receptor-dependent effects in regulating the synergism, indicating the efficacy of the combination in treating the heterogenous subtypes of BC.

While drug-induced over-expression of TS is described as the major mechanism of chemoresistance hampering the success of 5-FU chemotherapy (38), the down-regulation of TS significantly augmented the anticancer potential of 5-FU, due to the attenuation in its primary target (9, 10). The present study also demonstrated that treatment with 5-FU-alone induces a considerable elevation in the TS levels, which was significantly down-regulated by curcumin pretreatment improving the therapeutic index of 5-FU.

Numerous studies have associated NF-κB over-expression with therapeutic resistance against 5-FU (16, 39), and inhibition of NF-κB activity with the enhancement of 5-FU cytotoxicity (40, 41). The constitutively higher levels of NF-κB have been reported as an intrinsic feature of cell lines resistant to TS inhibitors (40). Meanwhile, the efficacy of curcumin in down-regulating the oncogenic activity of NF-κB is well documented (15, 24). Moreover, a recent study has shown that curcumin analogs effectively inhibit ectopically induced NF-κB activation and subsequent over-expression of TS in colorectal cancer cells (42).

In concordance with above reports, the present study too revealed a drug-induced activation of NF-κB, which was radically down-regulated by curcumin. In addition to TS and NF-κB, constitutive and drug-induced activation of Akt and MAPKs have been implicated in neoplastic cell proliferation, survival, and resistance (14, 15, 43). Numerous studies have shown the modulatory effects of curcumin on molecular pathways including Akt and MAPKs (20, 24). Our results also validate this property of curcumin to inhibit 5-FU-induced activation of these survival signals in the murine tumor samples treated with the combination. Among the various molecules studied, even though 5-FU-induced up-regulation of NF-κB, MAPKs and Akt were down-regulated by curcumin, they do not have any direct impact or regulatory role on the mode of action of the combination. The critical role of TS in this context was unraveled using MDA-MB-231^TS-^ xenograft model, where curcumin was ineffective in inducing a synergistic antitumor effect with 5-FU.

Studies have revealed that the therapeutic efficacy of 5-FU is greatly enhanced in the presence of a TS cofactor (44–46), which prompted us to hypothesize a cofactor-like role for curcumin. Molecular docking studies revealed the ability of curcumin to bind TS with an affinity shown towards the TS cofactor-binding site, rather than the substrate-binding site, where 5-FU binds. This in silico data explains a probable mechanism behind the curcumin-mediated increase in the therapeutic index of 5-FU. Our modeling analysis comparing the affinity of curcumin with that of LY231514 (pemetrexed), a multitargeted antifolate used in the treatment of advanced breast cancer, indicated a superior affinity of curcumin over LY231514 towards TS cofactor-binding site. Curcumin enhances the anticancer effect of the well-known antifolate, methotrexate, against gastric cancer as per the studies (47, 48) and an *in silico* analysis shows curcumin binding to dihydrofolate reductase (DHFR) with comparable binding energy to that of methotrexate (49). Assembling these observations we predict an antifolate-like activity for curcumin, similar to that of pemetrexed, which may significantly enhance the therapeutic efficacy of 5-FU by inhibiting TS activity highlighting curcumin as a potential alternative for antifolate adjuvant chemotherapy with 5-FU.

Even though 5-FU is not a key drug in the current clinical scenario of breast cancer chemotherapy, it is used either alone or in combination with other drugs for the successful treatment and management of metastatic breast cancer (50). Recent reports also demonstrate that 5-FU can be used either alone or in combination with other drugs to treat triple negative breast cancer effectively (51, 52). Though numerous studies have demonstrated the anticancer potential of curcumin against various cancers, none of the clinical trials conducted using this phytochemical has generated substantial outputs. This is due to the reason that the maximum attainable serum concentration of curcumin is around 5µM, a concentration that is not sufficient to induce cytotoxicity in cancer cells of any origin. In the present study, we have demonstrated that 5µM curcumin is sufficient to down-regulate 5-FU-induced survival signals, which eventually leads to the up-regulation of TS that develops chemoresistance in TNBC cells. Our treatment modality results in a drastic reduction of 5FU dosage, minimizing the toxicity and cost of chemotherapy. Moreover, this is the first study, which highlights TS as a promising clinical target of curcumin-mediated chemosensitization of BC to 5-FU chemotherapy. Taken together, our study identifies a novel strategy with minimum side effects, which significantly improves the efficacy of 5FU chemotherapy in a receptor -independent mode and hence could be effectively used for treating TNBC patients, who have limited therapeutic options.

## Conclusions

The present study is the first preclinical evaluation of the therapeutic efficacy of the 5-FU and curcumin combination against BC. Moreover, based on the recent observations where therapies that attenuate *de novo* pyrimidine synthesis sensitize drug-resistant TNBC to chemotherapy (53), we put forward a novel therapeutic strategy that treats BC irrespective of their receptor status. Though we proclaim the effectiveness of the combination with collective pre-clinical evidence, it necessitates clinical validation in patients with different molecular subtypes of BC, with special emphasis on TNBC. TS has been reported to be the major factor sustaining the de-differentiated status of triple-negative breast cancers (54). A very recent study demonstrating a pharmacologically safe, intravenous co-administration of curcumin with paclitaxel in patients with advanced metastatic BC, points out an excellent future prospective for combinatorial regimen employing curcumin with conventional chemotherapeutics (55). Currently, we have initiated studies on evaluating the efficacy of 5-FU-curcumin combination against breast cancer stem cell population and primary breast cancer cells isolated from patients of divergent receptor status.

## Data Availability Statement

The original contributions presented in the study are included in the article/**Supplementary Material**. Further inquiries can be directed to the corresponding author.

## Ethics Statement

The animal study was reviewed and approved by Institutional Animal Ethics Committee, Rajiv Gandhi Centre for Biotechnology (IAEC/230/RUBY), University of Florida Institutional Animal Care and Use Committee #RD3585.

## Author Contributions

Conception and design: RA. Development of methodology: HN, MZ-K, and RA. Acquisition of data (Animal experiments, histopathology, immunohistochemistry, protein expression studies and statistical analysis): HN, NA, VV, VL, VA, AS, SM, TH, AK, NN, and MG. Molecular docking studies and computational analysis: AA and SC. Histopathology and immunohistochemistry analysis and verification: SS. Analysis and interpretation of data: HN, NA, BV, SB, AA, SC, MZ-K, and RA. Writing, review, and/or revision of the manuscript: HN, NA, VL, SB, MG, MZ-K, and RA. Study supervision: RA and MZ-K. All authors contributed to the article and approved the submitted version.

## Funding

Financial support for this work has been provided by the Council of Scientific & In-dustrial Research (to HN), Government of India and Department of Biotechnology, Govern-ment of India (to RA). This work was supported in part by Public Health Service grant R01 CA-188132 (to MZ-K) from the National Institute of Health and the Gatorade Trust through funds distributed by the University of Florida, Department of Medicine (to MZ-K).

## Conflict of Interest

The authors declare that the research was conducted in the absence of any commercial or financial relationships that could be construed as a potential conflict of interest.

## Supplementary Material

Supplementary Figure 1
**(A, B)** Graphs showing the percentage of apoptotic cells in different treatment groups upon H&E staining and TUNEL staining of the tumor sections, respectively. Data represent two independent sets of experiments and results are shown as the mean ± S.D. P-values were calculated with one-way ANOVA. ***P-values ≤0.001, **P-values ≤0.01 and *P-values ≤0.05. **(C, D)** Graphs showing a comparison of average tumor volume of MDA-MB-231^TS^ and MDA-MB-231^TS-^ xenografts, respectively from start date of drug treatment till the completion of treatment. Significant reduction in tumor volume is observed in animals bearing MDA-MB-231^TS^ xenografts, upon treatment with combination while no significant reduction in tumor volume is observed in animals bearing MDA-MB-231^TS-^ xenografts. Data represent two independent sets of experiments and results are shown as the mean ± S.D. P-values were calculated with one-way ANOVA. ***P-values ≤0.001, **P-values ≤0.01 and *P-values ≤0.05; ns represents non-significance.

Supplementary Table 1 The intensity of protein bands of respective proteins in corresponding immunoblot figures analysed by ImageJ software. C1, C2, C3-Control samples 1, 2, 3;Cu1, Cu2, Cu3- Curcumin treated samples1, 2, 3;F1, F2, F3- 5-FU treated samples 1, 2, 3;F+C1, F+C2, F+C3- Combination treated samples 1, 2, 3.

## Abbreviations

BC, breast cancer; TNBC, triple negative breast cancer; 5-FU, 5-Fluorouracil; TS, thymidylate synthase; NF-κB, nuclear factor κB; MAPK, mitogen-activated protein kinase; HER-2, human epidermal growth factor receptor-2; TUNEL, terminal deoxy-nucleotidyl transferase-mediated nick end labeling.
