# Corrigendum: Targeting thymidylate synthase enhances the chemosensitivity of triple-negative breast cancer towards 5-FU-based combinatorial therapy

**DOI:** 10.3389/fonc.2023.1302413

**Published:** 2023-12-15

**Authors:** Nair Hariprasad Haritha, Akbar Nawab, Vinod Vijayakurup, Nikhil Ponnoor Anto, Vijayasteltar B. Liju, Vijai V. Alex, Areekkara Nisthul Amrutha, Sreekumar U. Aiswarya, Mundanattu Swetha, Balachandran S. Vinod, Sankar Sundaram, Maria V. Guijarro, Thomas Herlevich, Archana Krishna, Nesteena K. Nestory, Smitha V. Bava, Chittalakkottu Sadasivan, Maria Zajac-Kaye, Ruby John Anto

**Affiliations:** ^1^ Division of Cancer Research, Rajiv Gandhi Centre for Biotechnology, Thiruvananthapuram, India; ^2^ Department of Anatomy and Cell Biology, Cancer and Genetics Research Complex, University of Florida, Gainesville, FL, United States; ^3^ The Shraga Segal Department of Microbiology, Immunology and Genetics, Faculty of Health Sciences, Ben-Gurion University of the Negev, Beer Sheva, Israel; ^4^ Department of Biotechnology and Microbiology, Kannur University, Kannur, India; ^5^ Department of Biotechnology, University of Calicut, Malappuram, India; ^6^ Department of Pathology, Government Medical College, Kottayam, India

**Keywords:** breast cancer, thymidylate synthase, chemoresistance, chemosensitization, curcumin, 5-FU

In the published article, there was an error in **Figure 5A**(i) and **Figure 5D**(ii) as published. The image of excised tumor of animal treated with curcumin that is present in Figure 2A was wrongly placed as image of excised tumor in the control group in **Figure 5A**(i). In **Figure 5D**(ii), an error happened during the cropping and placing of the IHC images. The IHC image depicting the expression status of TS in curcumin-treated tumor samples of MDA-MB-231 TS^-ve^ panel was accidentally duplicated and the same image was placed as IHC image depicting the expression status of p-p65 in curcumin-treated tumor samples of MDA-MB-231 TS^-ve^ panel. We have rectified this mistake in the revised **Figure 5** and the duplicated images have been replaced by the correct ones. The corrected **Figure 5** and its caption appear below.

**Figure 5:**
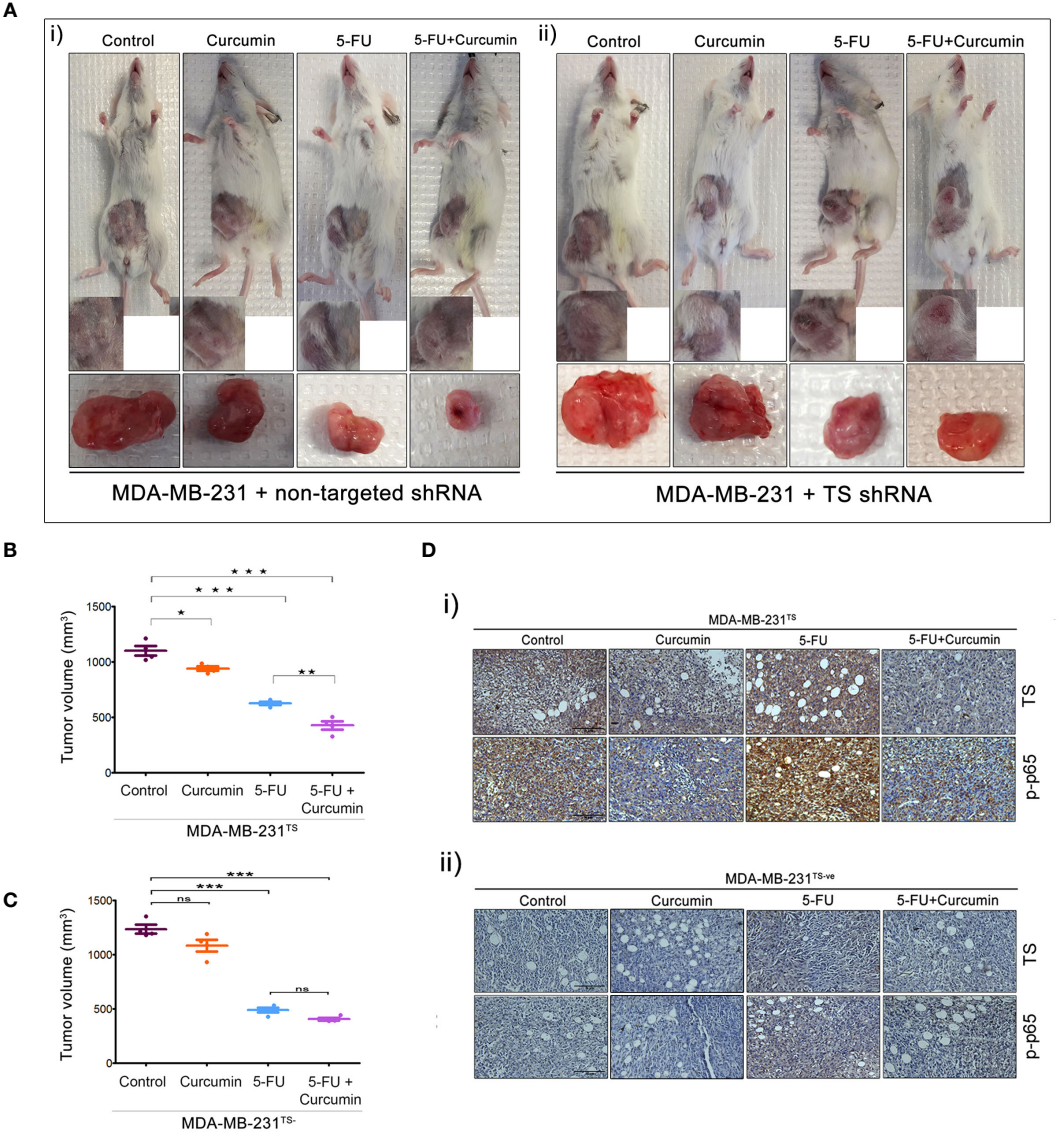
The role of TS in regulating the synergism of 5-FU and curcumin. [**A** (i, ii)] Representative images of animals from each experimental group, bearing tumor produced by orthotopic injection of both sets of transduced cells and tumors excised showed a reduction in tumor size upon completion of treatment. **(B, C)** Graphs showing a comparison of tumor volume of MDA-MB-231^TS^ and MDA-MB-231^TS-^ xenografts, respectively upon completion of treatment. Significant reduction in tumor volume is observed in animals bearing MDA-MB-231^TS^ xenografts, upon treatment with combination while no significant reduction in tumor volume is observed in animals bearing MDA-MB-231^TS-^ xenografts. Data represent two independent sets of experiments and results are shown as the mean ± S.D. P-values were calculated with one-way ANOVA. ***P-values ≤0.001, **P-values ≤0.01 and *P-values ≤0.05; ns represents non-significance. [**D** (i, ii)] Immunohistochemical analysis of expression status of TS and p65 sub-unit of NF-κB in different treatment groups of MDA-MB-231^TS^ and MDA-MB-231^TS-^ xenografts, respectively.

The authors apologize for this error and state that this does not change the scientific conclusions of the article in any way. The original article has been updated.

