# Supplementary figures and images for: Targeting Thymidylate Synthase Enhances the Chemosensitivity of Triple-Negative Breast Cancer Towards 5-FU-Based Combinatorial Therapy
Source: Front Oncol. 2021 Jul 15;11:656804. doi: 10.3389/fonc.2021.656804 (PMC8320437; doi:10.3389/fonc.2021.656804)

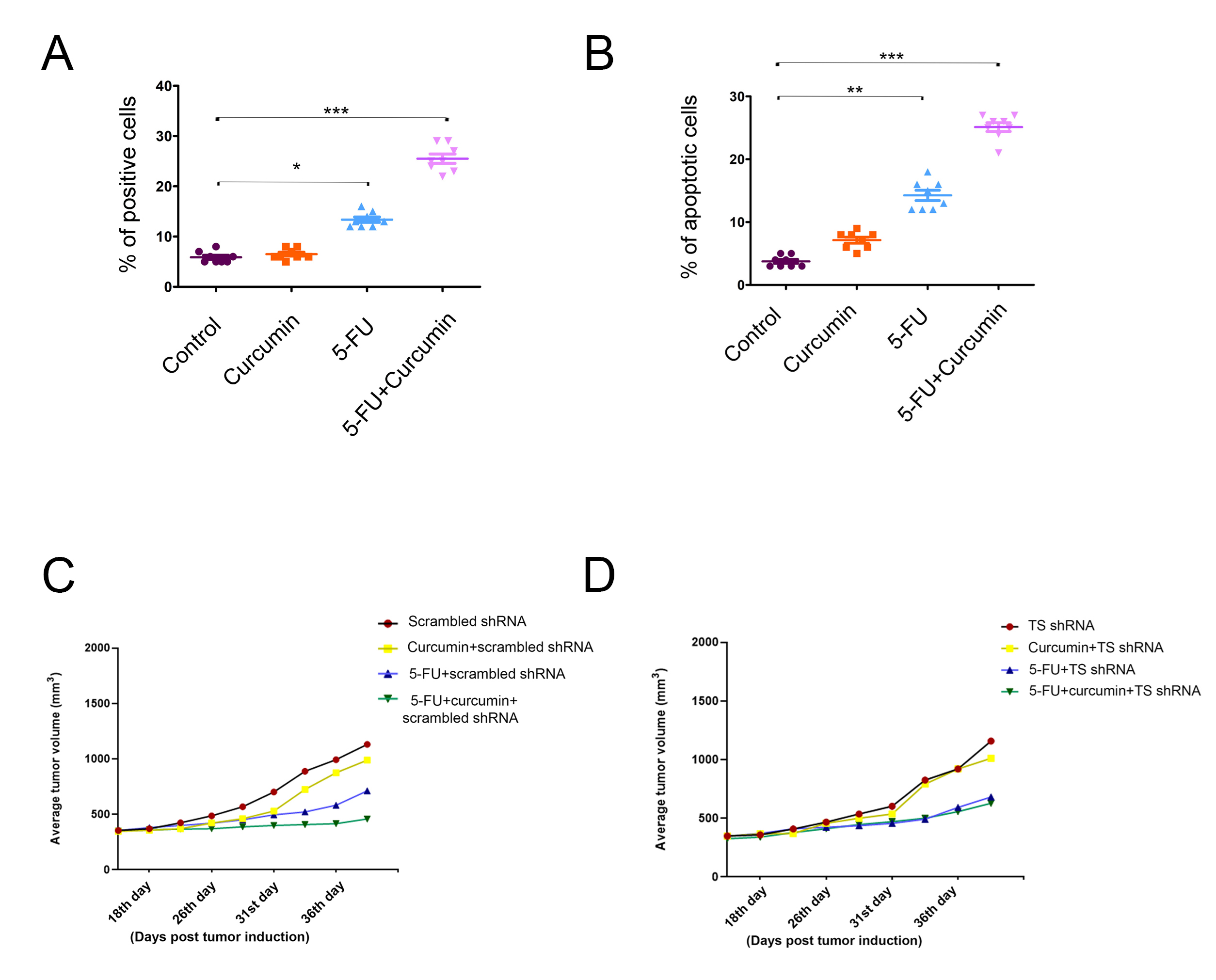

Supplement: Supplementary Figure 1 — (A, B) Graphs showing the percentage of apoptotic cells in different treatment groups upon H&E staining and TUNEL staining of the tumor sections, respectively. Data represent two independent sets of experiments and results are shown as the mean ± S.D. P-values were calculated with one-way ANOVA. ***P-values ≤0.001, **P-values ≤0.01 and *P-values ≤0.05. (C, D) Graphs showing a comparison of average tumor volume of MDA-MB-231TS and MDA-MB-231TS- xenografts, respectively from start date of drug treatment till the completion of treatment. Significant reduction in tumor volume is observed in animals bearing MDA-MB-231TS xenografts, upon treatment with combination while no significant reduction in tumor volume is observed in animals bearing MDA-MB-231TS- xenografts. Data represent two independent sets of experiments and results are shown as the mean ± S.D. P-values were calculated with one-way ANOVA. ***P-values ≤0.001, **P-values ≤0.01 and *P-values ≤0.05; ns represents non-significance. [file Image_1.jpeg]
