# Supplementary material for: Targeting Thymidylate Synthase Enhances the Chemosensitivity of Triple-Negative Breast Cancer Towards 5-FU-Based Combinatorial Therapy
Source: Front Oncol. 2021 Jul 15;11:656804. doi: 10.3389/fonc.2021.656804 (PMC8320437; doi:10.3389/fonc.2021.656804)
Supplement: Supplementary Table 1 — The intensity of protein bands of respective proteins in corresponding immunoblot figures analysed by ImageJ software. C1, C2, C3-Control samples 1, 2, 3;Cu1, Cu2, Cu3- Curcumin treated samples1, 2, 3;F1, F2, F3- 5-FU treated samples 1, 2, 3;F+C1, F+C2, F+C3- Combination treated samples 1, 2, 3. [file Table_1.doc]

| **Figure 2E** | **C1** | **C2** | **C3** | **Cu1** | **Cu2** | **Cu3** | **F1** | **F2** | **F3** | **F+C1** | **F+C2** | **F+C3** |
| --- | --- | --- | --- | --- | --- | --- | --- | --- | --- | --- | --- | --- |
| Caspase 8 | 1.0 | 0.7 | 1.0 | 0.9 | 0.6 | 0.7 | 0.8 | 1.1 | 1.0 | 0.6 | 0.3 | 0.9 |
| Caspase 7 | 0.7 | 1.0 | 0.64 | 0.84 | 0.56 | 0.42 | 0.46 | 0.24 | 0.45 | 0.04 | 0.12 | 0.38 |
| **Figure 2F** | **C1** | **C2** | **C3** | **Cu1** | **Cu2** | **Cu3** | **F1** | **F2** | **F3** | **F+C1** | **F+C2** | **F+C3** |
| PARP cleavage | 1.0 | 0.47 | 0.04 | 0.29 | 0.18 | 0.38 | 0.17 | 0.13 | 0.44 | 1.71 | 1.59 | 2.09 |
| **Figure 3A** | **C1** | **C2** | **C3** | **Cu1** | **Cu2** | **Cu3** | **F1** | **F2** | **F3** | **F+C1** | **F+C2** | **F+C3** |
| TS | 1.0 | 0.85 | 0.33 | 0.39 | 0.01 | 0.14 | 1.96 | 2.52 | 2.30 | 1.81 | 0.04 | 0.01 |
| p-p65 | 0.1 | 0.4 | 1.0 | 0.31 | 0.97 | 0.52 | 0.84 | 0.7 | 0.95 | 0.48 | 0.4 | 0.23 |
| p-IKK | 0.9 | 0.0 | 0.3 | 2.10 | 3.5 | 4.7 | 6.0 | 2.3 | 4.4 | 3.8 | 1.0 | 0.8 |
| **Figure 3B** | **C1** | **C2** | **C3** | **Cu1** | **Cu2** | **Cu3** | **F1** | **F2** | **F3** | **F+C1** | **F+C2** | **F+C3** |
| XIAP | 0.1 | 0.8 | 1.7 | 0.1 | 0.4 | 1.0 | 0.7 | 0.6 | 1.3 | 0.0 | 0.0 | 0.3 |
| c-IAP1 | 0.67 | 1.0 | 0.7 | 1.55 | 0.26 | 1.29 | 1.91 | 2.16 | 1.9 | 0.9 | 0.13 | 0.01 |
| Bcl2 | 1.0 | 1.4 | 0.1 | 1.1 | 0.4 | 1.0 | 1.5 | 1.8 | 2.6 | 0.9 | 0.8 | 0.6 |
| **Figure 3C** | **C1** | **C2** | **C3** | **Cu1** | **Cu2** | **Cu3** | **F1** | **F2** | **F3** | **F+C1** | **F+C2** | **F+C3** |
| p-Akt | 1.6 | 1.0 | 0.5 | 0.4 | 1.0 | 0.1 | 1.1 | 0.2 | 1.4 | 0.5 | 0.0 | 0.4 |
| p-p38 | 0.96 | 1.00 | 1.92 | 1.59 | 1.46 | 0.93 | 1.03 | 0.06 | 0.92 | 0.78 | 0.77 | 0.70 |
| p-JNK | 3.04 | 1.00 | 0.51 | 2.10 | 2.03 | 1.84 | 1.95 | 0.66 | 2.26 | 0.06 | 0.05 | 0.80 |
| **Figure 3E** | **C1** | **C2** | **C3** | **Cu1** | **Cu2** | **Cu3** | **F1** | **F2** | **F3** | **F+C1** | **F+C2** | **F+C3** |
| ABCG2 | 0.83 | 1.00 | 0.75 | 1.62 | 1.21 | 1.42 | 1.91 | 1.68 | 1.43 | 1.63 | 1.32 | 1.35 |
| VEGF | 1.23 | 1.00 | 1.17 | 1.23 | 0.87 | 0.53 | 0.78 | 0.65 | 0.67 | 0.09 | 0.04 | 0.15 |
| **Figure 4B** | **48h shRNA 64** | **48h**  **Cntrl shRNA** | **48h**  **MDA-MB-231** | **24h shRNA 64** | **24h**  **Cntrl shRNA** | **24h**  **MDA-MB-231** |  |  |  |  |  |  |
| TS | 0.01 | 1.35 | 1.00 | 0.31 | 0.93 | 1.00 |  |  |  |  |  |  |
|  |  |  |  |  |  |  |  |  |  |  |  |  |

Supplementary table 1
